# Task sharing in an interprofessional medication management program – a survey of general practitioners and community pharmacists

**DOI:** 10.1186/s12913-022-08378-4

**Published:** 2022-08-06

**Authors:** Robert Moecker, Marina Weissenborn, Anja Klingenberg, Lucas Wirbka, Andreas Fuchs, Christiane Eickhoff, Uta Mueller, Martin Schulz, Petra Kaufmann-Kolle, ARMIN Study Group, Anja Auerbach, Dorit Braun, Catharina Doehler, Susanne Donner, Stefan Fink, Jona Frasch, Christine Honscha, Urs Dieter Kuhn, Mike Maetzler, Ulf Maywald, Andreas D. Meid, Anke Moeckel, Carmen Ruff, Felicitas Stoll, Kathrin Wagner, Walter E. Haefeli, Hanna M. Seidling

**Affiliations:** 1 Cooperation Unit Clinical Pharmacy, University of Heidelberg, Im Neuenheimer Feld 410, 69120 Heidelberg, Germany; 2 Department of Clinical Pharmacology and Pharmacoepidemiology, Heidelberg University Hospital, Im Neuenheimer Feld 410, 69120 Heidelberg, Germany; 3 aQua Institute for Applied Quality Improvement and Research in Health Care, Maschmühlenweg 8–10, 37073 Göttingen, Germany; 4 AOK PLUS – Die Gesundheitskasse, Sternplatz 7, 01067 Dresden, Germany; 5 Department of Medicine, ABDA – Federal Union of German Associations of Pharmacists, Heidestraße 7, 10557 Berlin, Germany; 6 Institute of Pharmacy, Freie Universität Berlin, Kelchstraße 31, 12169 Berlin, Germany

**Keywords:** Interprofessional medication management, Medication review, Primary care, Task sharing, Survey

## Abstract

**Background:**

Pharmacist-led medication review and medication management programs (MMP) are well-known strategies to improve medication safety and effectiveness. If performed interprofessionally, outcomes might even improve. However, little is known about task sharing in interprofessional MMP, in which general practitioners (GPs) and community pharmacists (CPs) collaboratively perform medication reviews and continuously follow-up on patients with designated medical and pharmaceutical tasks, respectively. In 2016, ARMIN (Arzneimittelinitiative Sachsen-Thüringen) an interprofessional MMP was launched in two German federal states, Saxony and Thuringia. The aim of this study was to understand how GPs and CPs share tasks in MMP when reviewing the patients’ medication.

**Methods:**

This was a cross-sectional postal survey among GPs and CPs who participated in the MMP. Participants were asked who completed which MMP tasks, e.g., checking drug-drug interactions, dosing, and side effects. In total, 15 MMP tasks were surveyed using a 5-point Likert scale ranging from “I complete this task alone” to “GP/CP completes this task alone”. The study was conducted between 11/2020 and 04/2021. Data was analyzed using descriptive statistics.

**Results:**

In total, 114/165 (69.1%) GPs and 166/243 (68.3%) CPs returned a questionnaire. The majority of GPs and CPs reported (i) checking clinical parameters and medication overuse and underuse to be completed by GPs, (ii) checking storage conditions of drugs and initial compilation of the patient’s medication including brown bag review being mostly performed by CPs, and (iii) checking side-effects, non-adherence, and continuous updating of the medication list were carried out jointly. The responses differed most for problems with self-medication and adding and removing over-the-counter medicines from the medication list. In addition, the responses revealed that some MMP tasks were not sufficiently performed by either GPs or CPs.

**Conclusions:**

Both GPs’ and CPs’ expertise are needed to perform MMP as comprehensively as possible. Future studies should explore how GPs and CPs can complement each other in MMP most efficiently.

**Supplementary Information:**

The online version contains supplementary material available at 10.1186/s12913-022-08378-4.

## Background

Medication reviews (MR) are a well-known strategy to improve medication safety and effectiveness. They can solve drug-related problems (DRP), improve medication appropriateness, and clinical outcomes [1, 2]. If good interprofessional collaboration is in place, MR might be implemented more efficiently [3]. Furthermore, interprofessional collaboration between general practitioners (GPs) and community pharmacists (CPs) has positive effects on blood pressure or HbA_1c_values [4–9], among others, and possibly on asthma patients [10]. In addition, the integration of CPs in primary care teams shows positive effects on LDL-cholesterol levels, and appropriate medication use [11].

Although interprofessionalism can improve patient care, regular involvement of both GPs and CPs in MR is rare [12]. Studies that have investigated interprofessional MR and medication management in primary care have often only addressed how GPs and CPs interact at an organizational level in the provision of a service [13, 14]. The intervention usually started with GPs sending their patients to the pharmacy for a MR. The pharmacy then performed the MR and forwarded recommendations for action to the GP [15, 16]. Prior studies usually investigated the attitude of GPs towards the conduct of MR by CPs, whether the pharmacists’ recommendations for action were implemented by GPs, or how the cooperation between GPs and CPs can be successfully designed [17–21]. In contrast, little research has been done on interprofessional collaboration in medication management which comprises continuous care of the patient in a multidisciplinary team following a MR. In particular, little is known about how medication management tasks, such as checking of drug-drug interactions, duplicate medications, or guideline-adherence are shared between GPs and CPs [22].

In Germany, an interprofessional medication management program (MMP) was introduced in 2016 and implemented by CPs working in community pharmacies and GPs as part of ARMIN (“Arzneimittelinitiative Sachsen-Thüringen”), a project endorsed by professional associations and a statutory health insurance (SHI) fund (*AOK PLUS*). ARMIN was implemented in two federal states, Saxony and Thuringia [23]. In the MMP, patients signing up for the program chose both a GP and a community pharmacy that jointly supervised the patients’ drug therapy. Specific tasks and responsibilities were assigned to each professional group. Participating GPs should take care of the medical aspects, including guideline-directed medical therapy, while participating CPs should take care of the pharmaceutical aspects, including instructions on the correct use of medicines. Other tasks under the MMP included the review of over-the-counter (OTC) medicines and continuous updating of the medication list [24]. Both GPs and CPs receive about EUR 100 for the medication review (one-time reimbursement) and about EUR 25 for a follow-up (up to every three months).

The aim of this study was to investigate how the MMP was implemented by GPs and CPs in routine clinical practice by analyzing who performed which tasks in the MMP. In addition, we examined how different GP-CP pairs carried out their task sharing i.e., whether tasks were carried out by both health care professionals (HCP) or by neither HCP, to gain insights into how well or even how diversely GPs and CPs coordinate task sharing in MMP.

## Methods

Commissioned through the initiators of the ARMIN project, the study was conducted by the Department of Clinical Pharmacology and Pharmacoepidemiology at Heidelberg University Hospital together with the aQua Institute for Applied Quality Improvement and Research in Health Care (Göttingen, Germany). As part of the evaluation of the ARMIN project, participating GPs and CPs were pseudonymously surveyed about their experiences with the ARMIN project. The cross-sectional study was reported according to the Consensus-Based Checklist for Reporting of Survey Studies (CROSS) [25] (see Additional file 1).

Until the beginning of 2020, 15.8% (243/1536) of the CPs and 3.9% (165/4178) of the GPs in the federal states of Saxony and Thuringia had registered for the MMP service and enrolled at least one patient in the MMP.

### Questionnaire development

We developed a questionnaire that included nine sections: sociodemographic and general information, technology/software, implementation of the ARMIN program including MMP (see Additional file 2), impact of the ARMIN program on HCPs’ daily work, impact on non-ARMIN patients’ therapy and care, benefits, costs, cost–benefit ratio, and fulfillment of expectations and wishes. Response scales in the questionnaire mainly comprised Likert scales or single/multiple response options. The questionnaire was developed as a self-administered postal questionnaire. Organizations involved in the ARMIN project (see ARMIN study group) supported the questionnaire development.

If available, already validated questionnaires were used, such as two questionnaires to assess physician-pharmacist collaboration (ATCI; FICI) [26]. In addition, new sets of questions were developed for ARMIN MMP-specific workflows. The questionnaire was subjected to an internal quality assurance process to check comprehensibility, clarity, and unambiguity [27]. Then, it was piloted with GPs and CPs participating in ARMIN to ensure that the questionnaire is suitable for the target group. In the first round, five GPs and CPs each received the questionnaire via email or fax. All questions were piloted with the HCPs via videophone or phone using think-aloud and cognitive interviewing techniques. Adjustments, including the deletion of less relevant questions and the rewording of questions were iteratively incorporated into the questionnaire.

The survey procedure was tested in a second pilot round with five additional GPs and CPs each. The questionnaire was sent by regular mail, the participating HCPs filled in the questionnaires themselves, made comments on unclear questions and/or ambiguous answer options, and returned the questionnaire in a prepaid envelope. In a subsequent debriefing phone call, the HCPs’ comments were discussed and the adjustments made after the first round of piloting were reassessed. HCP considered the questionnaires to be easily understandable and clearly structured. As HCPs had no other important comments, the pilot phase was successfully completed.

### Participants and recruitment

The target population of the survey, all GPs and CPs who participated in the MMP of at least one patient by September 1, 2020, was invited by the SHI fund to participate in the survey. Therefore, the AOK PLUS sent the questionnaire to 165 GPs and 243 CPs in November 2020. Concurrently, the Associations of Statutory Health Insurance Physicians and the State Associations of Pharmacists informed their members about the survey in order to increase the response rate. A postal reminder was sent in December 2020 and an additional telephone reminder was issued at the end of January 2021. Both reminders were provided by the AOK PLUS and only sent to non-responders.

### Data collection

The data collection took place between November 2020 and April 2021. Participants sent the questionnaire to the aQua Institute that extracted and digitized the responses using validated automatic data digitization systems. Then, aQua Institute sent the data sets to the Department of Clinical Pharmacology and Pharmacoepidemiology.

The data collection was pseudonymized by the SHI fund. Because only the SHI fund had access to the pseudonyms, aQua Institute and the Department of Clinical Pharmacology and Pharmacoepidemiology at the Heidelberg University Hospital collected de facto anonymous questionnaire data. Conversely, the SHI fund had no access to individual questionnaire data sets.

In this paper, we only report on the section “task sharing in the ARMIN MMP”. Participants were asked to rank task sharing for 15 MMP tasks with regard to the GP or CP with whom they supervised most of their patients. MMP tasks are: Initial compilation of the patient's medication including brown bag review, checking drug-drug interactions, duplicate medications, dosing, administration times, inadequate dosage forms, storage condition of drugs, side effects, non-adherence, problems regarding self-medication, clinical parameters, and medication overuse and underuse as well as explanation of and handing out the medication list, adding and removing OTC medicines from the medication list, and continuous updating of the medication list.

### Data analyses

In a descriptive analysis, relative frequencies of GPs’ and CPs’ answers were calculated. Furthermore, to calculate mean Likert scores, Likert responses of GPs were coded as follows: “I complete this task alone” = 100%, “I complete this task with CP’s support” = 75%, “Equally” = 50%, “CP completes this task with my support” = 25%, or “CP completes this task alone” = 0%. Likert responses of CPs were coded in an analogous way: “I complete this task alone” = 100%, “I complete this task with GP’s support” = 75%, “Equally” = 50%, “GP completes this task with my support” = 25%, or “GP completes this task alone” = 0%.

In a sub-analysis, together with the SHI fund, we identified distinct GP-CP pairs who collaborated the most regarding the number of common patients in the MMP. These GP-CP pairs were identified in order to describe task sharing for distinct GP-CP pairs which were unambiguously identifiable. In contrast to the descriptive analysis, in which different perceptions of all GPs and CPs were analyzed, in this sub-analysis we aimed to identify MMP tasks that were insufficiently or sufficiently coordinated by distinct GP-CP pairs. The coordination of MMP tasks between both HCPs was operationalized by summing up the GPs’ and CPs’ part in performing each task. For example, if the CPs responded “GP completes this task with my support” (25%) and the corresponding GP responded “CP completes this task alone” (0%), a sum score would be 25% indicating that coordination for this task could be improved. Sum scores were analyzed using three categories, i.e., < 100%, = 100%, and > 100%. While a sum score of 100% could indicate a well-coordinated delivery of a task, a lower or higher sum score could be an indicator that coordination of collaboration within a GP-CP pair needs improvement.

IBM SPSS Statistics for Windows, Version 28.0 (IBM Corp., Armonk, NY, USA) and R-Studio 1.4.1717 (R Foundation for Statistical Computing, Vienna, Austria) were used for data entry and analysis. Respondents with > 25% missing answers in the entire questionnaire and > 50% missing answers in the sections of interest for the planned analysis, i.e., the key section ‘task sharing in MMP’ and the key section ‘communication with the other HCPs’, were excluded from the analysis. Limits and key sections were discussed among the researchers. A combination of multiple criteria was found appropriate to exclude non-responders without eliminating to much data. Results were analyzed using descriptive statistics. Differences between GPs’ and CPs’ Likert responses were analyzed using Mann–Whitney-U test. The test was two-sided with an alpha level of 0.05.

## Results

### Participants

Of 165 GPs and 243 CPs approached, 114 (response rate 69.1%) and 166 (68.3%) returned the questionnaire, respectively. Of these, 2/114 (1.8%) and 3/166 (1.8%) questionnaires were excluded due to incomplete responses. Ultimately, we included 112 (67.9%) questionnaires from GPs and 163 (67.1%) questionnaires from CPs in the descriptive analysis (Table 1).

**Table 1:** Characteristics of the respondents

Characteristic	GPs (*n* = 112)	CPs (*n* = 163)
Age [years] Median (IQR)	52 (45–60)	44 (37–52)
Work experience in a family practice/ community pharmacy [years] Median (IQR)	13 (8–26)	18 (9.5–25)
Sex [n (%)]		
Male	59 (52.7)	40 (24.5)
Female	52 (46.4)	121 (74.2)
Region [n (%)]		
Rural	57 (50.9)	84 (51.5)
Urban	54 (48.2)	79 (48.5)
Position [n (%)]		
Owner	105 (93.8)	83 (50.9)
Employee	6 (5.4)	80 (49.1)
Type of GP’s medical practice [n (%)]		
Single practice	84 (75.0)	NA
Joint practice	23 (20.5)	NA
Medical service center	3 (2.7)	NA
Other	1 (0.9)	NA
Issued medication lists before participation in the MMP [n (%)]	111 (99.1)	35 (21.5)
Using local software	110/111 (99.1)	25/35 (71.4)
Using other software (e.g., MS Office)	1/111 (0.9)	10/35 28.6)
Conducted medication reviews before participation in the MMP [n (%)]	80 (71.4)	81 (49.7)
Alone	59/80 (73.8)	59/81 (72.8)
With consultation of the respective other HCP	21/80 (26.3)	22/81 (27.2)

*HCP* health care professional, *MMP* medication management program. Due to rounding or missing responses, percentages may not always add up to 100%

### Descriptive analysis of the perception of MMP tasks in the CP and GP population

Responses of GPs and CPs corresponded well for typically medical and pharmaceutical tasks: While GPs stated that they mainly complete (“I complete this task alone” and “I complete this task with CP’s support”) checking clinical parameters (n = 106, 94.7%), medication overuse and underuse (*n* = 91, 81.3%), and dosing (*n* = 83, 74.1%), CPs stated that tasks for which they mainly relied on GPs (“GP completes this task with my support” and “GP completes this task alone”) were checking clinical parameters (*n* = 144, 88.3%), medication overuse and underuse (*n* = 100, 61.3%), and dosing (*n* = 64, 39.3%). Conversely, CPs stated that tasks they mainly complete (“I complete this task alone” and “I complete this task with GP’s support”) are checking storage condition of drugs (*n* = 154, 94.5%), initial compilation of the patient’s medication (*n* = 148, 90.8%), and checking administration times (*n* = 118, 72.4%). GPs stated that tasks for which they mainly relied on CPs (“CP completes this task with my support” and “CP completes this task alone”) were checking storage condition of drugs (*n* = 84, 75.0%), initial compilation of patient’s medication (*n* = 61, 54.5%), and checking inadequate dosage form (*n* = 38, 33.9%).

On the other hand, HCPs see a shared responsibility for many MMP tasks. GPs most often responded “Equally” for drug-drug-interactions (*n* = 60, 53.6%), duplicate medication (*n* = 49, 43.8%), administration times (*n* = 36, 32.1%), inadequate dosage forms (*n* = 36, 32.1%), side effects (*n* = 49, 43.8%), non-adherence (*n* = 35, 31.3%), problems regarding self-medication (*n* = 37, 33.0%), adding and removing OTC medicines from the medication list (*n* = 44, 39.3%), and continuous updating of the medication list (*n* = 46, 41.1%). CPs most often responded “Equally” for dosing (*n* = 46, 28.2%), side effects (*n* = 61, 37.4%), non-adherence (*n* = 52, 31.9%), explanation of and handing out the medication list (*n* = 55, 33.7%), and continuous updating of the medication list (*n* = 82, 50.3%). Likert responses from GPs and CPs for all MMP tasks are shown in Table 2.

**Table 2:** Relative frequencies [%] of GPs’ (*n* = 112) and CPs’ (*n* = 163) responses regarding task sharing in MMP. The mode is printed in bold

Task	HCP	GP completes this task alone	GP completes this task with CP’s support	Equally	CP completes this task with GP's support	CP completes this task alone	Don't know	Missing	*p*-value
Initial compilation of the patient's medication (incl. brown bag review)	GP	13.4	8.9	20.5	21.4	**33.0**	0.0	2.7	< 0.001
	CP	1.2	1.2	6.1	9.8	**81.0**	0.6	0.0	
Drug-drug interactions	GP	8.0	9.8	**53.6**	15.2	13.4	0.0	0.0	< 0.001
	CP	0.6	1.8	22.1	21.5	**50.9**	1.2	1.8	
Duplicate medications	GP	12.5	17.9	**43.8**	12.5	9.8	2.7	0.9	< 0.001
	CP	0.0	1.8	25.2	20.9	**47.9**	3.1	1.2	
Dosing	GP	**43.8**	30.4	20.5	3.6	0.9	0.0	0.9	< 0.001
	CP	13.5	25.8	**28.2**	13.5	16.0	1.2	1.8	
Administration times (e.g. taking before meals)	GP	18.8	22.3	**32.1**	18.8	8.0	0.0	0.0	< 0.001
	CP	1.2	4.3	19.0	27.0	**45.4**	1.8	1.2	
Inadequate dosage forms	GP	12.5	17.0	**32.1**	26.8	7.1	3.6	0.9	< 0.001
	CP	2.5	5.5	20.2	24.5	**36.8**	8.0	2.5	
Storage condition of drugs	GP	0.0	3.6	17.0	31.3	**43.8**	4.5	0.0	< 0.001
	CP	0.0	0.6	1.2	14.7	**79.8**	3.1	0.6	
Side effects	GP	13.4	29.5	**43.8**	9.8	2.7	0.9	0.0	< 0.001
	CP	0.6	6.1	**37.4**	24.5	25.8	4.9	0.6	
Non-adherence	GP	29.5	28.6	**31.3**	4.5	1.8	2.7	1.8	< 0.001
	CP	4.3	9.8	**31.9**	21.5	19.6	12.3	0.6	
Problems regarding self-medication	GP	17.0	18.8	**33.0**	19.6	9.8	1.8	0.0	< 0.001
	CP	0.6	0.0	7.4	24.5	**66.3**	1.2	0.0	
Clinical parameters (e.g. eGFR, HbA1c)	GP	**79.5**	15.2	2.7	0.9	0.0	0.0	1.8	0.846
	CP	**73.0**	15.3	1.8	0.6	0.6	8.6	0.0	
Medication overuse and underuse	GP	**53.6**	27.7	17.0	0.0	1.8	0.0	0.0	0.016
	CP	**34.4**	27.0	17.8	4.3	2.5	12.9	1.2	
Explanation of and handing out the medication list	GP	**44.6**	20.5	22.3	4.5	6.3	0.0	1.8	< 0.001
	CP	16.0	11.7	**33.7**	14.1	22.1	2.5	0.0	
Adding and removing OTC medicines from the medication list	GP	26.8	13.4	**39.3**	12.5	6.3	0.0	1.8	< 0.001
	CP	3.1	3.7	17.2	20.9	**51.5**	3.7	0.0	
Continuous updating of the medication list	GP	29.5	25.0	**41.1**	1.8	0.9	1.8	0.0	< 0.001
	CP	1.8	3.1	**50.3**	17.8	23.9	1.8	1.2	

*CP* community pharmacists, *eGFR* estimated glomerular filtration rate, *GP* general practitioner, *HbA1c* hemoglobin A1c, *HCP* health care professional, *OTC* over-the-counter

**Fig. 1:**
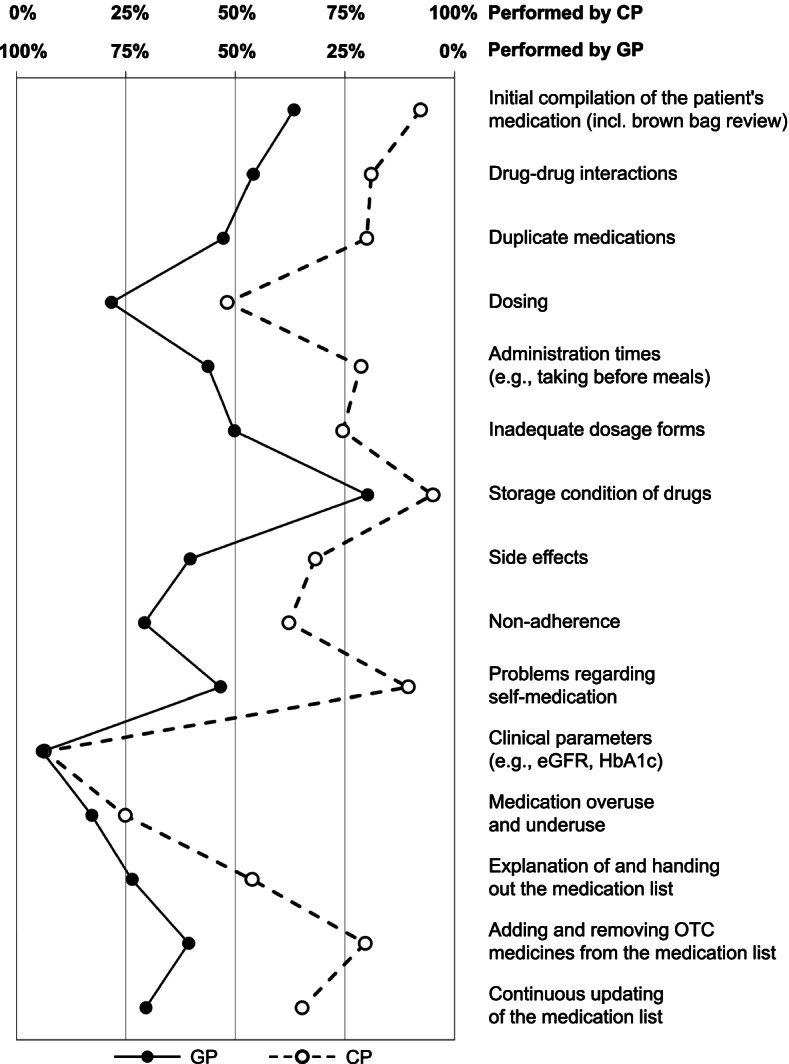
Mean Likert scores of GPs (*n* = 112) and CPs (*n* = 163) responses regarding task sharing in MMP

Results of mean Likert scores showed similar results (Fig. 1). GPs and CPs have a very similar perception of who completes the checking of clinical parameters and of medication overuse and underuse. On the other side, there is a considerable difference for tasks such as checking of problems regarding self-medication and adding and removing OTC medicines from the medication list. In general, GPs and CPs show a similar, yet shifted response pattern.

### Sub-analysis of MMP task sharing in distinct GP-CP pairs

Of 112 GPs and 163 CPs, 51 unique CP-GP pairs were identified. The GPs have been participating for 4.5 years and CPs for 4.25 years (median; based on time from first enrolment to the point of data collection (end of 2020)). The GP-CP pairs provided medication management for a total of 1623 patients (median: 21 patients, IQR: 11–52). Those pairs showed optimal coordination (sum score = 100%) for four tasks (median, IQR 3–6). However, many pairs had at least one MMP task with a lower sum score (sum score < 100%: *n* = 37, 72.5%). All pairs had at least for one MMP task a higher sum score (sum score > 100%: *n* = 51, 100%).

**Fig. 2:**
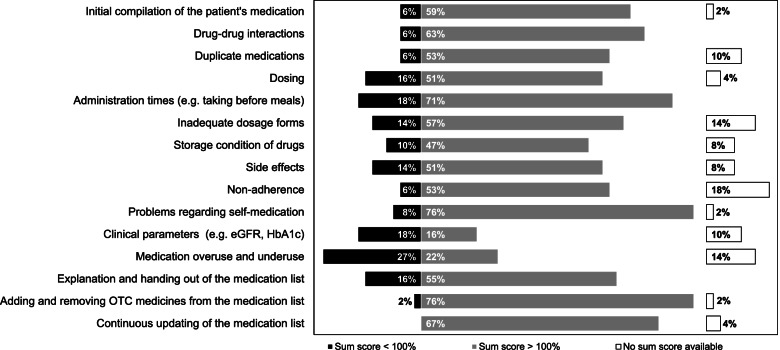
Relative frequencies of distinct GP-CP pairs (*n* = 51) showing a sum score of < 100% (indicator of low mutual recognition) and > 100% in MMP tasks (indicator of high mutual recognition); sum score = 100 not shown

Regarding the different MMP tasks, a sum score < 100% was observed for 14 (27.5%) pairs in checking medication overuse and underuse, 9 (17.6%) for clinical parameters, and 9 (17.6%) for administration times (Fig. 2). A sum score > 100% was obtained for 39 (76.5%) pairs in checking for problems regarding self-medication and for adding and removing OTC medicines from the medication list, 36 (70.6%) for administration times.

Regarding the number of mutual patients for whom a GP-CP pair provided medication management, no trend was observed that pairs with a higher number of mutual patients (as an indicator of their level of involvement in ARMIN MMP) had fewer MMP task with sum scores < 100% (or > 100%).

## Discussion

Interprofessional MMP is a complex intervention that requires good coordination and collaboration between GPs and CPs [28, 29]. This study provides information on which MMP tasks GPs and CPs provide and how they perceive their collaboration in interprofessional medication management at the operational level.

GPs perceive that they mainly completed medical tasks such as checking dosing, clinical parameters, and medication overuse and underuse. CPs perceive that they mainly completed pharmaceutical tasks such as initial compilation of the patient’s medication and problems regarding self-medication. However, for many tasks we observed a difference in how GPs and CPs perceived task sharing. For all tasks, except checking clinical parameters, CPs see themselves more responsible than GPs see CPs responsible (and vice versa). GPs’ and CPs’ opinions diverge most concerning problems regarding self-medication, adding and removing OTC medicines from the medication list, and the continuous updating of the medication list. In contrast, GPs and CPs agreed most that checking clinical parameters and medication overuse and underuse are medical tasks and initial compilation of the patient’s medication including brown bag review and storage condition of drugs are pharmaceutical tasks. Furthermore, both GPs and CPs considered checking potential drug-drug interactions rather a pharmaceutical than a medical task.

These findings align with the initially planned and designated tasks in the ARMIN MMP, which defined that GPs should complete medical tasks, such as checking guideline-adherence, and CPs should complete pharmaceutical tasks, such as the initial compilation of the patient's medication including brown bag review [30]. Although some flexibility in MMP task sharing is needed and has to be well-coordinated, the concept of the interprofessional MMP in ARMIN might serve as a blueprint for how tasks between GPs and CPs could be shared in future MMP, at least in Germany [31]. In addition, the involvement of CPs in MMP could respond to the desire of GPs to be supported by trained pharmacists when GPs are facing increased workloads due to growing number of patients with chronic conditions [32].

Furthermore, we examined how GP-CP pairs recognize sharing of MMP tasks. A sum score < 100% shows less mutual recognition than a sum score > 100%. We hypothesize that a sum score < 100% could indicate that a given task is not sufficiently addressed by a GP-CP pair and, therefore, needs special attention in interprofessional medication management programs. A sum score > 100% could indicate high mutual recognition or it might suggest that a particular task is not being performed efficiently, because both HCPs are investing more time and resources than might be necessary. If one might want to take a deeper look in how medication management is performed, deviating or irregular sum scores might give an indication on where it is worth taking a closer look. This might be true when comparing sum scores of different tasks within one GP-CP pair but also when comparing sum scores of distinct tasks. In this study we investigated 15 tasks of which 11 tasks are distinct checks performed during medication reviews. Obviously, there are tasks where a sum score > 100% is likely, because both HCPs perform the same task as part of their professional role. For example, checking drug-drug interaction or non-adherence in duplicate might be reasonable because those tasks require comprehensive information gathering and intense patient counselling (ideally by both HCPs). Other tasks such as checking clinical parameters and the storage conditions of drugs, should be clearly attributed to one HCP only to increase efficiency. Hence, also given a potential recall bias, deviant sum scores might be an indicator that the process of the interprofessional medication management could be improved. Also, a sum score < 100% most likely indicates that the process of the interprofessional medication management could be improved. For example, the check of medication overuse and underuse should be addressed and could be improved because more than a quarter of the GP-CP pairs had a sum score < 100%. This could be addressed by suggesting available effective and validated tools to detect overuse and underuse [33, 34]. The importance of taking action here is underlined by the fact that drug use without indication and untreated/undertreated indication ranks among the top four DRPs in large ambulatory populations [35]. More than 15% of the GP-CP pairs had sum scores < 100% for clinical parameters, administration times, dosing, and explanation of and handing out the medication list. This gap of coordination could potentially lead to a gap in performed DRP checks in MMP and subsequently even impair medication safety. As an example, inappropriately low dosing of direct oral anticoagulants in atrial fibrillation does not only result in lower plasma concentrations but also in more severe strokes and worse functional outcomes [36]. However, for most MMP tasks, most GP-CP pairs had sum scores > 100%, possibly indicating that both HCPs overlap in performing MMP tasks. Still, it has to be further investigated if the observed overlap corresponds with different perceptions of both HCPs, intentional double-checking, or redundancy (or if high sum scores are primarily the result of social desirability bias).

Two further findings deserve closer examination. First, collaborating GPs and CPs do not always seem to have the same idea of who performs which tasks and to what extent, a finding that has been reported previously [37]. In some cases, each HCP of a pair indicated that they were doing a task mainly (or even alone) – without knowing or acknowledging that the corresponding collaborating HCP was also involved. Therefore, communication between both HCPs about exactly this content would be important to minimize the workload in this GP-CP pair and to optimize the content of the joint care. Second, there may be discussion about which tasks could be primarily the responsibility of one HCP and which tasks should be performed by both GPs and CPs. Nonetheless, well-coordinated task sharing and acknowledgement of what had already been accomplished by the other HCP could possibly limit redundancy in both workflows and, therefore, reduce workload and ultimately save time and money. However, this trade-off must be made very carefully, as, with fewer double checks, medication errors could go undetected more easily, especially with high risk drugs [38].

Therefore, both HCPs should be in close and continuous coordination with each other. On the one hand, to avoid potential gaps in medication safety, and on the other hand, to save resources.

### Limitations

This study has several limitations. First, we conducted a cross-sectional study, preventing any analysis of potential trends over time such as fewer cases of potential gaps in the provision of MMP with increasing time of HCPs’ participation. However, we analyzed potential gaps – that is, GPs and CPs underperforming a MMP task, depending on the number of patients shared by a GP-CP pair as an indicator of their level of involvement in ARMIN MMP. Furthermore, we observed many MMP tasks with sum scores > 100%. This result could be due to the fact that HCPs chose a response indicating more responsibility and effort (social-desirability bias). Also, participants might have remembered only the last few patients (recall bias). However, as patients usually visit GPs and CPs regularly (at least every 3 months) recall bias was probably low. Overall, the participation of CPs and GPs in the MMP was rather low, limiting the generalizability of our findings. However, a high response rate of approximately 70% for both HCPs suggests that the survey results are representative for the study population of MMP participants. Another limitation is, that this was an explorative study that can only propose hypotheses about the potential reasons for and impact of insufficient coordination in MMP on medication safety and effectiveness. Future studies should investigate which tasks need overlapping and in which proportions they should be carried out by which HCP, so that the best possible drug therapy results for the patient.

## Conclusions

In general, GPs and CPs participating in the ARMIN program shared most of the tasks in the interprofessional MMP, as envisaged in the original concept, and many of their tasks complemented each other. In some tasks, however, the allocation was less clear, which might have led to tasks either not being carried out sufficiently or being carried out in duplicate. In projects where tasks overlap, ways should be found to promote interprofessional communication in order to save resources and close gaps in care.

## Supplementary Information

**Additional file 1:** 

**Additional file 2:** 

## Data Availability

The datasets generated and/or analyzed during the current study are not publicly available because the contract between the sponsor (SHI fund) and the contractor (researchers) does not allow the publication or sharing of data. Data are however available from the authors upon reasonable request and with permission of the SHI fund.

## Abbreviations

ARMIN: Arzneimittelinitiative Sachsen-Thüringen
CP: Community pharmacist
eGFR: Estimated glomerular filtration rate
GP: General practitioner
HbA1c: Hemoglobin A1c
HCP: Healthcare professional
MMP: Medication management program
MR: Medication review
OTC: Over-the-counter
SHI: Statutory health insurance
