# Supplementary material for: Task sharing in an interprofessional medication management program – a survey of general practitioners and community pharmacists
Source: BMC Health Serv Res. 2022 Aug 6;22:1005. doi: 10.1186/s12913-022-08378-4 (PMC9356506; doi:10.1186/s12913-022-08378-4)
Supplement: Supplementary file 1 — Additional file 1: [file 12913_2022_8378_MOESM1_ESM.pdf]

Task sharing in an interprofessional medication management program – a survey of general practitioners and community pharmacists

Robert Moecker[1,2], Marina Weissenborn[1,2], Anja Klingenberg[3], Lucas Wirbka[2], Andreas Fuchs[4], Christiane Eickhoff[5], Uta Mueller[5], Martin Schulz[5,6], Petra Kaufmann-Kolle[3], ARMIN Study Group[7], Walter E. Haefeli[1,2], Hanna M. Seidling[1,2]

CROSS-Checklist

Sharma A, Minh Duc NT, Luu Lam Thang T, et al. A Consensus-Based Checklist for Reporting of Survey Studies (CROSS). J. Gen. Intern. Med. 2021;36:3179-3187.

**Title and abstract**			**Reported on lines**	**Text**
**Title and abstract**	1a	State the word "survey" along with a commonly used term in title or abstract to introduce the study's design	title page	Task sharing in an interprofessional medication management program – a survey of general practitioners and community pharmacists
	1b	Provide an informative summary in the abstract, covering background, objectives, methods, findings/results, interpretation/discussion, and conclusions.	See abstract	Abstract
**Introduction**				
**Background**	2	Provide a background about the rationale of study, what has been previously done, and why this survey is needed.	51-58	Prior studies usually investigated the attitude of GPs towards the conduct of MR by CPs, whether the pharmacists' recommendations for action were implemented by GPs, or how the cooperation between GPs and CPs can be successfully designed.17-21 In contrast, little research has been done on interprofessional collaboration in medication management which comprises continuous care of the patient in a multidisciplinary team following a MR. In particular, little is known about how medication management tasks, such as checking of drug-drug interactions, duplicate medications, or guideline-adherence are shared between GPs and CPs.
**Purpose/aim**	3	Identify specific purposes, aims, goals, or objectives of the study.	72-76	The aim of this study was to investigate how the MMP was implemented by GPs and CPs in routine clinical practice by analyzing who performed which tasks in the MMP. In addition, we examined how different GP-CP pairs carried out their task sharing i.e., whether tasks were carried out by both health care professionals (HCP) or by neither HCP, to gain insights into how well or even how diversely GPs and CPs coordinate task sharing in MMP.
**Methods**				
**Study design**	4	Specify the study design in the "Methods" section with a commonly used term (e.g., cross-sectional or longitudinal).	83-84	The cross-sectional study was reported according to…
**Data collection methods**	5a	Describe the questionnaire (e.g., number of sections, number of questions, number and names of instruments used).	89-93<br><br>134-135	We developed a questionnaire that included nine sections: sociodemographic and general information, technology/software, implementation of the ARMIN program including MMP (see Additional file 2), impact of the ARMIN program on HCPs' daily work, impact on non-ARMIN patients' therapy and care, benefits, costs, cost-benefit ratio, and fulfillment of expectations and wishes.<br><br>Participants were asked to rank task sharing for 15 MMP tasks …
	5b	Describe all questionnaire instruments that were used in the survey to measure particular concepts. Report target population, reported validity and reliability information, scoring/classification procedure, and reference links (if any).	95-98	The questionnaire was developed as a self-administered postal questionnaire. Organizations involved in the ARMIN project (see ARMIN study group) supported the questionnaire development. If available, already validated questionnaires were used …

				Comment: As many questions aimed at assessing project-related parameters, such as who performed which tasks, how did the workflow change etc., we could not use existing tools because specific questionnaire for program evaluation were needed. Results of ATCI and FICI were not reported. Hence, no psychometric parameters were reported.
	5c	Provide information on pretesting of the questionnaire, if performed (in the article or in an online supplement). Report the method of pretesting, number of times questionnaire was pre-tested, number and demographics of participants used for pretesting, and the level of similarity of demographics between pre-testing participants and sample population.	100-114	Then, it was piloted with GPs and CPs participating in ARMIN to ensure that the questionnaire is suitable for the target group. In the first round, five GPs and CPs each received the questionnaire via email or fax. All questions were piloted with the HCPs via videophone or phone using think-aloud and cognitive interviewing techniques. Adjustments, including the deletion of less relevant questions and the rewording of questions were iteratively incorporated into the questionnaire.<br>The survey procedure was tested in a second pilot round with five additional GPs and CPs each. The questionnaire was sent by regular mail, the participating HCPs filled in the questionnaires themselves, made comments on unclear questions and/or ambiguous answer options, and returned the questionnaire in a prepaid envelope. In a subsequent debriefing phone call, the HCPs' comments were discussed and the adjustments made after the first round of piloting were reassessed. HCP considered the questionnaires to be easily understandable and clearly structured. As HCPs had no other important comments, the pilot phase was successfully completed.<br><br>Comment: Demographic data, such as age or sex, of participants in pretests were not collected. However, Participants were from the same two federal states, were also GPs and CPs, were also involved in the ARMIN project.
	5d	Questionnaire, if possible, should be fully provided (in the article, or as appendices or as an online supplement).	89-91	We developed a questionnaire that included nine sections: sociodemographic and general information, technology/software, implementation of the ARMIN program including MMP (see Additional file 2), (…)
**Sample characteristics**	6a	Describe the study population (i.e., background, locations, eligibility criteria for participant inclusion in survey, exclusion criteria).	116-118	The target population of the survey, all GPs and CPs who participated in the MMP of at least one patient by September 1, 2020, was invited by the SHI fund to participate in the survey. Therefore, the AOK PLUS sent the questionnaire to 165 GPs and 243 CPs in November 2020.
	6b	Describe the sampling techniques used (e.g., single stage or multistage sampling, simple random sampling, stratified sampling, cluster sampling, convenience sampling). Specify the locations of sample participants whenever clustered sampling was applied.	119-123	Therefore, the AOK PLUS sent the questionnaire to 165 GPs and 243 CPs in November 2020. Concurrently, the Associations of Statutory Health Insurance Physicians and the State Associations of Pharmacists informed their members about the conduction of the survey in order to increase the response rate. A postal reminder was sent in December 2020 and an additional telephone reminder was issued at the end of January 2021. Both reminders were provided by the AOK PLUS and only sent to non-responders.
	6c	Provide information on sample size, along with details of sample size calculation.	117-118	The target population of the survey, all GPs and CPs who participated in the MMP of at least one patient by September 1,

Robert Moecker[1,2], Marina Weissenborn[1,2], Anja Klingenberg[3], Lucas Wirbka[2], Andreas Fuchs[4], Christiane Eickhoff[5], Uta Mueller[5], Martin Schulz[5,6], Petra Kaufmann-Kolle[3], ARMIN Study Group[7], Walter E. Haefeli[1,2], Hanna M. Seidling[1,2]

				2020, was invited by the SHI fund to participate in the survey.<br><br>Comment: sample size calculation: n.a.
	6d	Describe how representative the sample is of the study population (or target population if possible), particularly for population-based surveys.	178-179	Of 165 GPs and 243 CPs approached, 114 (response rate 69.1%) and 166 (68.3%) returned the questionnaire, respectively.
**Survey administration**	7a	Provide information on modes of questionnaire administration, including the type and number of contacts, the location where the survey was conducted (e.g., outpatient room or by use of online tools, such as SurveyMonkey).	94-95<br><br>121-123	The questionnaire was developed as a self-administered postal questionnaire.<br><br>A postal reminder was sent in December 2020 and an additional telephone reminder was issued at the end of January 2021. Both reminders were provided by the AOK PLUS and only sent to non-responders.
	7b	Provide information of survey's time frame, such as periods of recruitment, exposure, and follow-up days.	125	The data collection took place between November 2020 and April 2021.
	7c	Provide information on the entry process: –>For non-web-based surveys, provide approaches to minimize human error in data entry. >For web-based surveys, provide approaches to prevent "multiple participation" of participants.	126-128	… that extracted and digitized the responses using validated automatic data digitization systems… sent the data sets to the …
**Study preparation**	8	Describe any preparation process before conducting the survey (e.g., interviewers' training process, advertising the survey).	119-121	Concurrently, the Associations of Statutory Health Insurance Physicians and the State Associations of Pharmacists informed their members about the conduction of the survey in order to increase the response rate.
**Ethical considerations**	9a	Provide information on ethical approval for the survey if obtained, including informed consent, institutional review board [IRB] approval, Helsinki declaration, and good clinical practice [GCP] declaration (as appropriate).	365-369	All HCPs participated voluntarily and gave their informed consent before being included in this study. The study was conducted in accordance with the current version of the Declaration of Helsinki. Ethical approvals were obtained from the responsible Ethics Committee of the Medical Faculty of Heidelberg University (reference no.: S-142/2019 and S-230/2019).
	9c	Provide information about survey anonymity and confidentiality and describe what mechanisms were used to protect unauthorized access.	129-133	The data collection was pseudonymized by the SHI fund. Because only the SHI fund had access to the pseudonyms, aQua Institute and the Department of Clinical Pharmacology and Pharmacoepidemiology at the Heidelberg University Hospital collected de facto anonymous questionnaire data. Conversely, the SHI fund had no access to individual questionnaire data sets.
**Statistical analysis**	10a	Describe statistical methods and analytical approach. Report the statistical software that was used for data analysis.	144-145	In a descriptive analysis, relative frequencies of GPs' and CPs' answers were calculated. Furthermore, to calculate mean Likert scores, Likert responses of GPs were coded as follows…
	10b	Report any modification of variables used in the analysis, along with reference (if available).	n.a.	n.a.
	10c	Report details about how missing data was handled. Include rate of missing items, missing data mechanism (i.e., missing completely at random [MCAR], missing at random [MAR], or missing not at random [MNAR]), and methods used to deal with missing data (e.g., multiple imputation).	172-174	Results were analyzed using descriptive statistics. Differences between GPs' and CPs' Likert responses were analyzed using Mann-Whitney-U test. The test was two-sided with an alpha level of 0.05.

Robert Moecker[1,2], Marina Weissenborn[1,2], Anja Klingenberg[3], Lucas Wirbka[2], Andreas Fuchs[4], Christiane Eickhoff[5], Uta Mueller[5], Martin Schulz[5,6], Petra Kaufmann-Kolle[3], ARMIN Study Group[7], Walter E. Haefeli[1,2], Hanna M. Seidling[1,2]

	10d	State how non-response error was addressed.	167-170	Respondents with > 25% missing answers in the entire questionnaire and > 50% missing answers in the sections of interest for the planned analysis, i.e., the key section 'task sharing in MMP' and the key section 'communication with the other HCPs', were excluded from the analysis.
	10e	For longitudinal surveys, state how loss to follow-up was addressed.	n.a.	n.a.
	10f	Indicate whether any methods such as weighting of items or propensity scores have been used to adjust for non-representativeness of the sample.	n.a.	n.a.
	10g	Describe any sensitivity analysis conducted.	n.a.	n.a.
**Results**				
**Respondent characteristics**	11a	Report numbers of individuals at each stage of the study. Consider using a flow diagram, if possible.	178-181	Of 165 GPs and 243 CPs approached, 114 (response rate 69.1%) and 166 (68.3%) returned the questionnaire, respectively. Of these, 2/114 (1.8%) and 3/166 (1.8%) questionnaires were excluded due to incomplete responses. Ultimately, we included 112 (67.9%) questionnaires from GPs and 163 (67.1%) questionnaires from CPs in the descriptive analysis
	11b	Provide reasons for non-participation at each stage, if possible.	n.a.	Comment: We could not collect data about reasons for non-participation because HCPs did not respond to the postal questionnaire.<br>However, when HCP were reminded via telephone, they reported lack of time, other priorities (COVID), and not actively participating in ARMIN anymore.
	11c	Report response rate, present the definition of response rate or the formula used to calculate response rate.	see 11a	Of 165 GPs and 243 CPs approached, 114 (response rate 69.1%) and 166 (68.3%) returned the questionnaire, respectively. Of these, 2/114 (1.8%) and 3/166 (1.8%) questionnaires were excluded due to incomplete responses. Ultimately, we included 112 (67.9%) questionnaires from GPs and 163 (67.1%) questionnaires from CPs in the descriptive analysis
	11d	Provide information to define how unique visitors are determined. Report number of unique visitors along with relevant proportions (e.g., view proportion, participation proportion, completion proportion).	116-118<br><br>125	The target population of the survey, all GPs and CPs who participated in the MMP of at least one patient by September 1, 2020, was invited by the SHI fund to participate in the survey.<br><br>The data collection was pseudonymized by the SHI fund.
**Descriptive results**	12	Provide characteristics of study participants, as well as information on potential confounders and assessed outcomes.	179	Table 1
**Main findings**	13a	Give unadjusted estimates and, if applicable, confounder-adjusted estimates long with 95% confidence intervals and p values.	209	Table 2
	13b	For multivariable analysis, provide information on the model building process, model fit statistics, and model assumptions (as appropriate).	n.a.	Comment: no model used in the analysis
	13c	Provide details about any sensitivity analysis performed. If there are considerable amount of missing data, report sensitivity analyses comparing the results of complete cases with that of the imputed dataset (if possible).	see 10g	n.a.
**Discussion**				

Robert Moecker[1,2], Marina Weissenborn[1,2], Anja Klingenberg[3], Lucas Wirbka[2], Andreas Fuchs[4], Christiane Eickhoff[5], Uta Mueller[5], Martin Schulz[5,6], Petra Kaufmann-Kolle[3], ARMIN Study Group[7], Walter E. Haefeli[1,2], Hanna M. Seidling[1,2]

**Limitations**	14	Discuss the limitations of the study, considering sources of potential biases and imprecisions, such as nonrepresentativeness of sample, study design, important uncontrolled confounders.	327-344	This study has several limitations. First, we conducted a cross-sectional study, preventing any analysis of potential trends over time such as fewer cases of potential gaps in the provision of MMP with increasing time of HCPs' participation. However, we analyzed potential gaps – that is, GPs and CPs underperforming a MMP task, depending on the number of patients shared by a GP-CP pair as an indicator of their level of involvement in ARMIN MMP. Furthermore, we observed many MMP tasks with sum scores > 100%. This result could be due to the fact that HCPs chose a response indicating more responsibility and effort (social-desirability bias). Also, participants might have remembered only the last few patients (recall bias). However, as patients usually visit GPs and CPs regularly (at least every 3 months) recall bias was probably low. Overall, the participation of CPs and GPs in the MMP was rather low, limiting the generalizability of our findings. However, a high response rate of approximately 70% for both HCPs suggests that the survey results are representative for the study population of MMP participants. Another limitation is, that this was an explorative study that can only propose hypotheses about the potential reasons for and impact of insufficient coordination in MMP on medication safety and effectiveness. Future studies should investigate which tasks need overlapping and in which proportions they should be carried out by which HCP, so that the best possible drug therapy results for the patient.
**Interpretations**	15	Give a cautious overall interpretation of results, based on potential biases and imprecisions and suggest areas for future research.	342-351	Future studies should investigate which tasks need overlapping and in which proportions they should be carried out by which HCP, so that the best possible drug therapy results for the patient.<br>Conclusion<br>In general, GPs and CPs participating in the ARMIN program shared most of the tasks in the interprofessional MMP, as envisaged in the original concept, and many of their tasks complemented each other. In some tasks, however, the allocation was less clear, which might have led to tasks either not being carried out sufficiently or being carried out in duplicate. In projects where tasks overlap, ways should be found to promote interprofessional communication in order to save resources and close gaps in care.
**Generalizability**	16	Discuss the external validity of the results.	see 14	Overall, the participation of CPs and GPs in the MMP was rather low, limiting the generalizability of our findings. However, a high response rate of approximately 70% for both HCPs suggests that the survey results are representative for the study population of MMP participants.
**Other sections**				
**Role of the funding source**	17	State whether any funding organization has had any roles in the survey's design, implementation, and analysis.	385-397	This work was funded by the SHI fund AOK PLUS, the ABDA – Federal Union of German Associations of Pharmacists, the Association of Statutory Health Insurance Physicians – Saxony, and the Association of Statutory Health Insurance Physicians – Thuringia.
**Conflict of interest**	18	Declare any potential conflict of interest.	379-383	RM, MW, LW, WEH, and HMS declare they have received funding from the AOK

Robert Moecker[1,2], Marina Weissenborn[1,2], Anja Klingenberg[3], Lucas Wirbka[2], Andreas Fuchs[4], Christiane Eickhoff[5], Uta Mueller[5], Martin Schulz[5,6], Petra Kaufmann-Kolle[3], ARMIN Study Group[7], Walter E. Haefeli[1,2], Hanna M. Seidling[1,2]

				PLUS. AK, PKK, and JF received funding from Department of Clinical Pharmacology and Pharmacoepidemiology. Furthermore, the following members of the ARMIN study group have contributed to program development and conduct: CE, UM, MS, AF, DB, UM, CD, MM, AA, UDK, AM, CH, SD, SF, KW.
**Acknowledgements**	19	Provide names of organizations/ persons that are acknowledged along with their contribution to the research.	401	The authors thank all GPs and CPs for their participation in this study.
